# The effects of motivational verbal phrases on isoinertial force production and sustained force output: a comparison of positive and negative conditions

**DOI:** 10.3389/fpsyg.2026.1852572

**Published:** 2026-09-10

**Authors:** Gökhan Recep Aydın, Murat Kasap

**Affiliations:** Department of Coach Education, Faculty of Sports Sciences, Bartın University, Bartın, Türkiye

**Keywords:** motivational verbal phrases, isoinertial force, positive condition, negative conditions, Borg RPE scale

## Abstract

**Introduction:**

This study aimed to compare the effects of positive and negative motivational verbal phrases applied during short-term maximal isoinertial squat exercise on force production, sustained force output, and perceived exercise difficulty.

**Methods:**

Forty healthy university students (20 women and 20 men) from the Faculty of Sports Sciences voluntarily participated in this randomized crossover study. Each participant completed a 30-s maximal isoinertial squat test under three experimental conditions—silent (control), positive, and negative motivational verbal phrases—across three separate sessions. Verbal stimuli were delivered at the onset of the test and every 5 s thereafter at a standardized sound intensity of 60–70 dB. Maximal force, average force, and perceived exertion (Borg RPE) were recorded. Due to violations of normality assumptions, data were analyzed using Friedman and Wilcoxon signed-rank tests with Holm–Bonferroni correction.

**Results:**

Both positive and negative motivational verbal phrase conditions resulted in significantly greater maximal and average force output compared with the silent condition (*p* < 0.001). Force output was significantly higher under the negative motivational verbal phrase condition than under the positive condition. However, perceived exercise difficulty was also significantly elevated during negative motivational verbal phrase exposure (*p* < 0.01). No significant sex-based differences were observed in responses to motivational verbal phrases.

**Discussion:**

These findings demonstrate that not only the presence but also the content and emotional valence of motivational verbal phrases play an important role in short-term maximal strength performance and perceptual responses. Negative motivational verbal phrases may enhance force output, albeit at the cost of increased perceived exertion, highlighting the need for deliberate and context-specific use of verbal strategies in performance testing and training environments.

## Introduction

Physical performance is not limited to muscle strength and metabolic capacity; rather, it is a multidimensional construct shaped by an individual’s psychological state, motivational processes, and environmental stimuli. In the field of sports and exercise science, increasing attention has been directed toward examining how external stimuli presented during exercise influence performance outcomes. In this context, it has been emphasized that applied research in exercise science should be structured in accordance with evidence-based approaches (Amonette et al., 2016). One such stimulus, referred to as motivational verbal phrases, encompasses brief, directive, and motivating verbal statements delivered by coaches or researchers during exercise (Amagliani et al., 2010; Midgley et al., 2018).

Previous studies have demonstrated that the effects of motivational verbal phrases may vary depending on the type of exercise performed, the participant’s training status, and the frequency, timing, and sound intensity of the verbal stimulus (Midgley et al., 2018; Belkhiria et al., 2018). It has been reported that verbal phrases delivered at frequent intervals and sufficient sound intensity during maximal exercise testing can significantly enhance performance (Amagliani et al., 2010; Andreacci et al., 2002). These findings suggest that performance outcomes are sensitive not only to the presence of verbal stimuli but also to contextual and individual factors (Engel et al., 2018). Accordingly, not only the content of motivational verbal phrases but also the manner in which they are delivered appears to be critical in determining their effects on performance.

Maximal exercise tests are widely employed to assess physiological capacity and to inform the prescription of exercise programs (Ross, 2003). To obtain valid results from such tests, participants must exert true maximal effort, as submaximal attempts may compromise test validity (Chitwood et al., 1997). For this reason, motivational verbal phrases are frequently used to facilitate maximal effort and are recommended in several standardized testing guidelines (Ross, 2003; American Thoracic Society, 2002; Halperin et al., 2015). Early investigations have reported that motivational verbal phrases can enhance maximal voluntary muscle activation and force production (McNair et al., 1996). Although evidence regarding performance enhancement through motivational verbal phrases during long duration maximal tests (particularly VO₂max assessments) is relatively consistent, findings related to their effects during short duration maximal strength tasks remain inconclusive.

The majority of existing research has focused on positive motivational verbal phrases (e.g., “Keep going” or “You can do it”), typically comparing these conditions with silent or control conditions (Lee et al., 2021; Hammami et al., 2023). In contrast, experimental studies examining the effects of negative motivational verbal phrases (e.g., “You are exhausted” or “You cannot do it”), which are commonly encountered in competitive environments yet largely neglected in the literature, remain scarce. Notably, Halperin et al. (2020) directly compared positive, negative, and no-feedback conditions during repeated maximal isometric force production in resistance-trained individuals. They reported that negative verbal feedback resulted in greater force production than both positive and no-feedback conditions, suggesting that negatively valenced verbal stimuli may acutely enhance force production under certain circumstances. However, their study focused on repeated isometric elbow-flexor contractions and assessed force production and electromyographic activity. Thus, it remains unclear whether similar effects occur during dynamic lower body exercise involving continuous concentric–eccentric actions and whether such responses are accompanied by changes in perceived exertion.

Nevertheless, even in endurance-based tasks, motivational verbal phrases have been shown to significantly enhance performance (Neto et al., 2015). Furthermore, insufficient standardization of verbal phrase protocols with respect to timing, frequency, and sound intensity has contributed to methodological heterogeneity and limited comparability across studies (Midgley et al., 2018). Negative motivational verbal phrases may also possess ecological validity, as they reflect realistic environmental stimuli that athletes are exposed to during competition through opponent behavior, crowd reactions, or negative feedback.

Isometric and isoinertial strength tests offer distinct advantages for evaluating the effects of motivational verbal phrases, as both maximal force output and the sustainability of force overtime can be objectively quantified. Previous research has demonstrated that motivational verbal phrases can increase maximal force and the rate of force development during isometric handgrip tasks (Belkhiria et al., 2018). However, these effects appear to vary according to individual characteristics and task specific demands (Engel et al., 2019). In particular, during short duration maximal strength tasks, motivational verbal phrases may modulate performance through alterations in arousal and neurohormonal responses mediated by the central nervous system (Aktop, 2008).

From a theoretical perspective, the Yerkes and Dodson (1908) posits an inverted U-relationship between arousal and performance, suggesting that moderate to high arousal can enhance performance on simple or well-learned tasks. Negatively verbal phrases may increase sympathetic nervous system activation, raising arousal levels and thereby facilitating maximal force production. Alternatively, according to Self-Determination Theory (Ryan and Deci, 2000), negative verbal feedback may threaten the need for competence, triggering an avoidance motivation that paradoxically increases acute effort expenditure, albeit at the potential cost of reduced intrinsic motivation over time.

Accordingly, the aim of the present study was to compare the effects of positive and negative motivational verbal phrases applied during a 30 s continuous concentric–eccentric isoinertial squat test on maximal force production, average force output, and perceived exercise difficulty (Borg RPE) (Borg, 1982). Verbal phrases were delivered every 5 s using predetermined statements at a standardized sound intensity of 60–70 dB. By extending previous findings on negative verbal feedback to a dynamic lower body isoinertial exercise model, the present study sought to determine whether the performance-enhancing effects observed during repeated isometric force production also emerge during continuous concentric–eccentric exercise, while additionally considering perceived exertion as a perceptual response.

In line with this objective, the following research hypotheses were tested:

*H1*: The application of negative motivational verbal phrases does not result in a statistically significant difference in the sustainability of maximal strength over time (i.e., the average force level achieved throughout the test duration) compared with positive motivational verbal phrases and silent control conditions.

*H2*: The application of positive motivational verbal phrases results in a statistically significant difference in the maximal force output achieved during the isoinertial squat test compared with the silent control condition.

*H3*: Perceived exertion (Borg RPE) is significantly higher under the negative motivational verbal phrases condition compared with positive motivational verbal phrases and silent control conditions.

Accordingly, a standardized isoinertial squat protocol was implemented under controlled laboratory conditions to objectively evaluate the effects of positive and negative motivational verbal phrases on short term maximal force production and sustained force output.

## Materials and methods

### Participants

A total of 40 students (20 males and 20 females; age: 20.95 ± 1.75 years; BMI: 22.97 ± 3.25 kg/m^2^) from the Faculty of Sports Sciences at Bartın University voluntarily participated in the study. Participants engaged in physical activity, including aerobic or anaerobic exercise, at least 2 days per week, were familiar with isoinertial (flywheel) ergometer-based strength training, and had no known auditory or orthopedic impairments. Written informed consent was obtained from all participants after they were fully informed about the study procedures, objectives, and potential risks. The study was approved by the Bartın University Ethics Committee (Approval No: 2025-SBB-0244) and was conducted in accordance with the Declaration of Helsinki.

### Participants were eligible for inclusion if they met the following criteria

Participants were eligible for inclusion if they voluntarily agreed to participate, were between 18 and 24 years of age, had a minimum of 5 years of active sports experience, were currently enrolled as students in the Faculty of Sports Sciences, had no injuries or health conditions that could preclude participation in isoinertial testing, and agreed to refrain from engaging in any additional training outside the test protocol during the study period. Participants who did not meet one or more of these inclusion criteria were excluded from the study.

### Randomization and condition concealment

The order in which participants completed the three experimental conditions silent control, encouraging verbal phrases, and discouraging verbal phrases was determined for each participant using a computer-generated randomization procedure.^1^ Each participant completed each condition once. The complete condition sequences are provided in Supplementary material S1.

Before each testing session, participants were not informed which verbal phrase condition they would encounter. They were also unaware of the complete sequence of conditions assigned across their sessions. Thus, the identity of the upcoming condition was concealed from participants until the test began. Participants were instructed not to discuss the content of the sessions or the verbal phrases they had encountered with other participants, thereby reducing the possibility of advance expectations arising from interparticipant information exchange.

Nevertheless, participants were not blinded to condition identity during the test. Once the verbal phrases protocol began, the presence, absence, and content of the phrases made the silent control, encouraging, and discouraging conditions readily identifiable. Accordingly, the procedure is described as concealment of the upcoming condition and session sequence rather than participant blinding. The researcher delivering the verbal phrases was necessarily aware of the condition being administered.

### Measured and observed parameters

The following primary outcome variables were assessed for each 30 s isoinertial maximal effort squat power protocol:

•Average force output produced during the test (kg).
•Maximal force output achieved during the test (kg).
•Perceived exertion, assessed immediately after each test session using the Borg Rating of Perceived Exertion (RPE) scale (6–20).

### Isoinertial force test protocol

Maximum concentric–eccentric strength measurements were performed using the Desmotec isoinertial exercise device using 30 s isoinertial maksimal effort power test protocol (Pininfarina, Italy). This gravity independent ergometer consists of a flywheel shaft mounted on a fixed frame, a belt system wrapped around the shaft, and a harness connected to the participant, enabling the generation and transmission of force. By means of interchangeable flywheel loads, the device allows force production capacities ranging from 0 to 500 kg.

A medium flywheel disc (diameter: 0.240 m; mass: 1.1 kg; moment of inertia: 0.008 kg·m^2^) was used for female participants, whereas a large flywheel disc (diameter: 0.285 m; mass: 1.9 kg; moment of inertia: 0.020 kg·m^2^) was used for male participants. The lower inertia disc was assigned to female participants and the higher inertia disc to male participants to accommodate the anticipated difference in absolute force production capacity and to permit both groups to perform continuous repetitions through the prescribed range of motion. The assigned disc remained unchanged for each participant across all three experimental conditions.

The system generates resistance proportional to the pulling force applied by the user, permitting lossless force transfer during both concentric and eccentric phases in terms of time and intensity. This bidirectional force transmission enables continuous and balanced mechanical loading throughout the exercise.

Participants performed the squat exercise using a specialized harness secured around the lumbar region. Each repetition began from a 90° knee flexion position, followed by an extension to 180° (full knee extension) during the concentric phase. Subsequently, resistance generated by the rotational inertia of the flywheel induced the eccentric squatting phase. Movements were executed at a 1:0:1 tempo, with continuous concentric and eccentric squat repetitions performed for a total duration of 30 s. Isoinertial systems are considered advantageous for evaluating both peak force production and sustained force output and are therefore frequently employed in studies investigating the acute effects of motivational verbal phrases (Rendos et al., 2019; Pacholek, 2024).

During each isoinertial test, maximal force (kg) and average force (kg) values were recorded in real time using the integrated software of the Desmotec device. These data were subsequently analyzed to assess both peak force production and the sustainability of force output across the test duration.

### Application procedure

**Figure 1:**
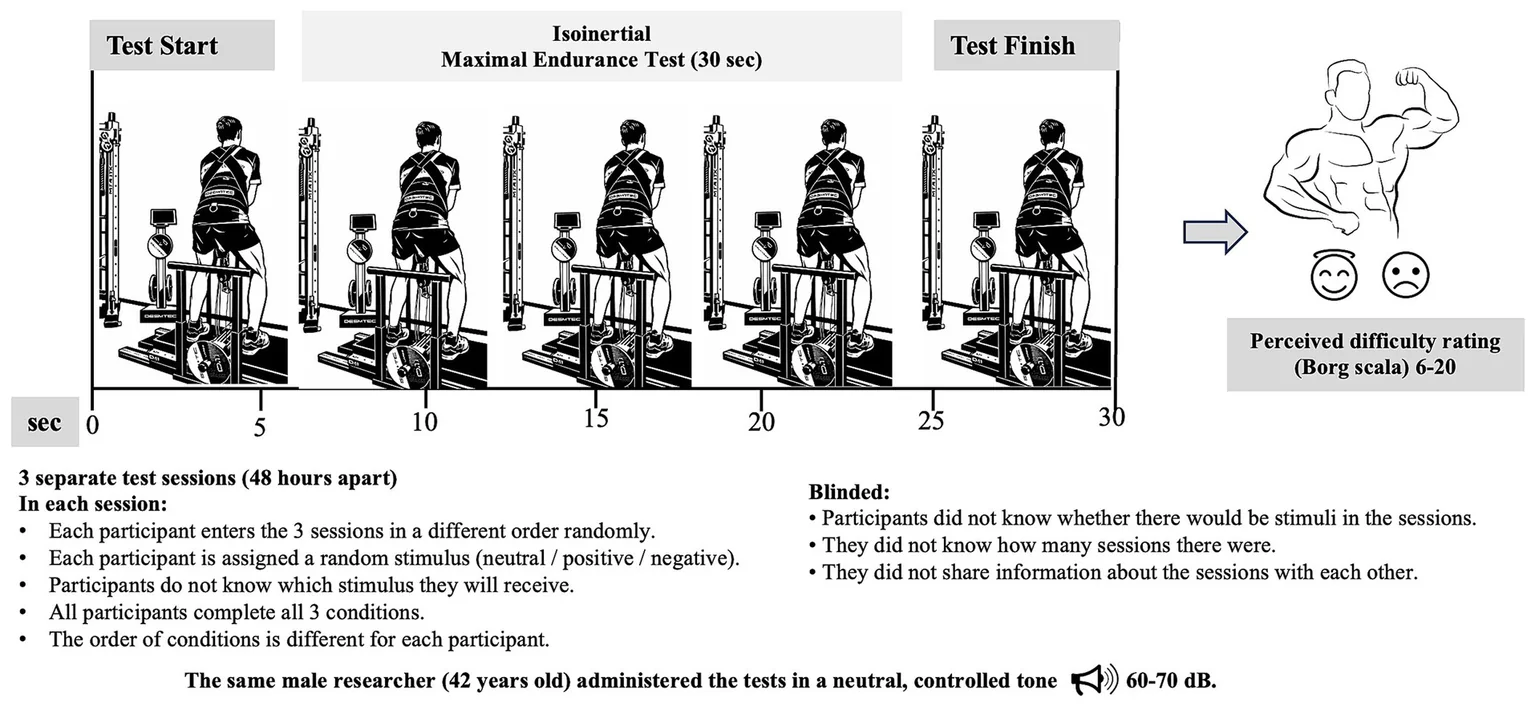
Test protocol layout.

A randomized crossover design was employed in the study. Each participant completed three separate testing sessions under different motivational verbal phrase conditions. Data collection was conducted between April 21 and April 30, 2025, following approval from the institutional ethics committee. The order of the sessions was randomized, and a minimum rest period of 48 h was allowed between sessions. This interval, together with the randomized condition order and the implementation of experimental conditions on separate days, was intended to minimize potential physiological and psychological carryover effects, consistent with previous randomized repeated measures research examining verbal stimuli during exercise (Adebiyi et al., 2025). All other experimental conditions were kept constant during the recovery period. This design is consistent with previous studies aiming to ensure between day reliability when examining the effects of motivational verbal phrases on performance (Engel et al., 2019). The overall protocol design is presented in Figure 1.

### Motivational verbal phrase protocol

The study employed three experimental conditions: silent control, encouraging verbal phrases, and discouraging verbal phrases. The order of these conditions was randomized and counterbalanced across participants. Participants were not informed of the complete condition sequence in advance and were asked not to discuss the content of the experimental sessions with other participants.

The verbal phrase protocol was developed to translate verbal interactions commonly encountered in resistance training environments into a controlled and experimentally reproducible procedure. In natural training settings, athletes may receive frequent but irregular encouraging statements from coaches or training partners when performing strenuous exercise. Conversely, discouraging or pressuring statements may sometimes be used in competitive contexts to undermine an opponent’s confidence or increase perceived pressure. To model these contrasting interactions under standardized experimental conditions, encouraging and discouraging verbal phrases were delivered according to an identical predetermined schedule.

Previous research has emphasized that the delivery characteristics of verbal encouragement including its timing, frequency, content, and intensity should be explicitly controlled and reported because variations in verbal delivery may influence exercise performance measurements (McNair et al., 1996; Midgley et al., 2018). However, these publications do not prescribe a 5-s phrase interval or a sound intensity range of 60–70 dB as empirically established thresholds. In the present study, these values were prespecified, study specific protocol parameters selected to convert the frequent but irregular verbal interactions observed in resistance training environments into a consistent and replicable experimental procedure.

**Figure 2:**
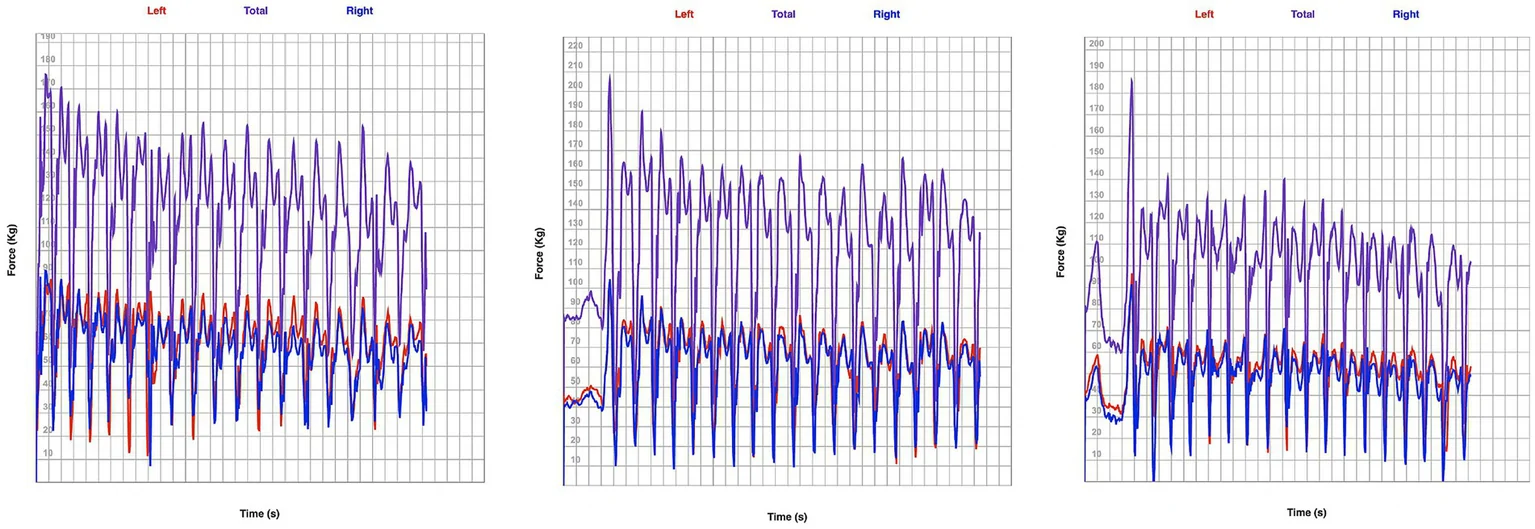
Representative force–time curves obtained during isoinertial testing under different verbal phrases (silent, positive, negative).

Verbal phrases were delivered at the onset of the 30-s maximal effort isoinertial squat test and every 5 s thereafter (i.e., at 0, 5, 10, 15, 20, and 25 s) (Figure 2). This schedule ensured that participants received the same number of phrases, at the same time points, in the encouraging and discouraging conditions. The 5-s interval should therefore be interpreted as an operational feature of the present protocol rather than an evidence based optimum or threshold. Its explicit specification enhances methodological reproducibility and provides a defined protocol for future studies comparing different verbal phrase frequencies.

Encouraging phrase consisted of supportive statements intended to reinforce confidence and continued effort, whereas discouraging phrase consisted of negatively valenced, challenging, and pressuring statements implying fatigue, poor performance, or inability to continue. The phrases were designed to experimentally represent contrasting verbal interactions that may occur in real world sporting and resistance training environments. Previous studies examining verbal encouragement and verbal feedback informed the broader conceptual development of the protocol (Pacholek, 2024; Puce et al., 2022); however, the exact phrases and delivery schedule used in the present study were specific to this experimental protocol.

All verbal phrases were delivered in Turkish, the participants’ native language, by the same male researcher (age 42 years), from a standardized position 1 m from the participant. The English versions presented in Table 1 are translations provided to allow international readers to understand the semantic and affective content of the original Turkish expressions. The translations were prepared using a forward and back translation procedure.

**Table 1:** Verbal stimuli used in silent (control), positive, and negative motivational conditions with Turkish originals, English translations, and delivery timing.

Time (sec.)	Silent	Positive (Turkish)	Positive (English)	Negative (Turkish)	Negative (English)
0	–	Yapabilirsin!	You can do it!	Yapamazsın!	You cannot do it!
5	–	Bas!	Push!	Çok kötü!	Too bad!
10	–	Devam et!	Keep going!	Devam edemezsin!	You cannot continue!
15	–	Basmaya devam et!	Keep pushing!	Tükeniyorsun!	You’re running out!
20	–	Hızlı!	Faster!	Yoruldun!	You’re tired!
25	–	Durma!	Do not stop!	Bitmiyor!	It does not end!

Positive phrases consisted of supportive, confidence-boosting words, while negative statements were oppressive and implied inadequacy.

Sound intensity was monitored during all verbal phrase sessions using a sound level meter (UNI-T UT353, UNI-Trend Technology, China). The meter was positioned 1 m from the researcher/participant. This range was used to ensure comparable auditory exposure across the encouraging and discouraging conditions and was a study specific standardization parameter rather than a literature derived threshold. The same researcher delivered all phrases using a controlled vocal manner to minimize between session variation in speaker characteristics. Participants could not view real time force data because the device display was turned away from them.

### Borg rating of perceived exertion

The Borg Rating of Perceived Exertion (RPE) scale is a widely used instrument for assessing an individual’s subjective perception of physical effort during exercise (Borg, 1982; Pescatello, 2014). The scale ranges from 6 (“no exertion at all”) to 20 (“maximal exertion”), with progressively increasing numerical values and verbal descriptors representing higher levels of perceived effort.

Immediately after completion of each 30-s isoinertial squat test, and before any discussion of performance or provision of force feedback, participants were shown a standardized printed A4 sheet displaying the complete Borg 6–20 RPE scale and its corresponding verbal descriptors. Participants were asked, “How hard did the exercise feel overall?”, and selected a single whole number value that best represented their overall perceived physical effort during the completed test. The researcher recorded the selected value directly on the participant’s data recording form without interpreting, modifying, or prompting the response. Thus, one RPE score was obtained for each participant in each of the three experimental conditions: silent control, encouraging verbal phrases, and discouraging verbal phrases.

### Anthropometric measurements

Participants’ height and body mass were measured using a calibrated SECA scale (Germany) equipped with an integrated stadiometer. The measurement accuracy of the device was ±0.1 cm for height and ±0.1 kg for body mass. Body mass index (BMI) was calculated using bioelectrical impedance analysis (BIA) with a TANITA BC420 device. A standardized electrical current of 50 kHz was applied through foot electrodes, and the measurements were recorded according to the manufacturer’s guidelines.

### Experimental design

During the week preceding the experimental period, participants completed a familiarization session in which all testing procedures, instruments, and exercise equipment were explained and practiced. Height, body mass, and body mass index (BMI) were assessed before the experimental testing sessions.

A randomized, counterbalanced crossover design was employed. Each participant completed three experimental testing sessions the silent control, encouraging verbal phrase, and discouraging verbal phrase conditions with a minimum interval of 48 h between consecutive sessions. The order of the three conditions was randomized and counterbalanced across participants. Detailed condition sequences are provided in Supplementary material S1.

Participants were not informed in advance of the complete sequence of experimental conditions or which condition they would encounter in an upcoming session. Thus, the upcoming condition was concealed until the test began. However, participants were not blinded to condition identity during testing because the presence, absence, and content of the verbal phrases made the assigned condition identifiable once the verbal phrase protocol commenced. Participants were instructed not to discuss the content of the testing sessions with other participants to reduce advance expectations and potential interparticipant information contamination.

Immediately after each 30-s isoinertial squat test, and before the provision of performance feedback, participants were shown a standardized printed A4 sheet displaying the complete Borg 6–20 Rating of Perceived Exertion (RPE) scale. They selected a single whole number value representing their overall perceived physical effort during the completed test. The selected value was recorded on the participant’s data recording form, resulting in one condition specific RPE score for each testing session.

To minimize potential circadian influences, all measurements were conducted on the same sports facility at the same time of day (between 08:00 and 10:00). To reduce injury risk and to prepare metabolic and neuromuscular systems for performance, participants completed a standardized 15-min warm up protocol prior to all testing sessions.

#### Warm-up protocol

The warm-up consisted of 5 min of low intensity cycling (50 W), 5 min of dynamic stretching (leg swings, torso twists, walking lunges), and 5 min of submaximal isoinertial squats (∼50% of perceived maximal effort).

### Statistical analysis

Descriptive statistics are presented as mean ± standard deviation (SD), together with minimum and maximum values. Data normality was assessed using the Shapiro–Wilk test, taking sample size into account. Normality analyses indicated that none of the dependent variables met the assumption of normal distribution across all motivational verbal phrase conditions and that perceived exercise difficulty (Borg RPE) represented an ordinal variable. Accordingly, non-parametric statistical methods were employed.

Nevertheless, although non-parametric tests were used in the final analyses due to violations of normality assumptions and the ordinal nature of the Borg RPE scale, the *a priori* power analysis was conducted based on the requirements of the experimental design.

The Friedman test, which is appropriate for repeated-measures designs, was used to determine differences among measurements obtained under the three motivational verbal phrase conditions (silent/neutral, positive, and negative). When significant effects were identified, pairwise comparisons were performed using the Wilcoxon signed rank test to determine which conditions differed. Holm–Bonferroni correction was applied to control for the potential inflation of Type I error due to multiple comparisons. Effect sizes were reported as Kendall’s W for Friedman tests and as r for pairwise comparisons. Kendall’s W values were interpreted as small (0.10), moderate (0.30), and large (0.50) effect sizes.

To examine the potential influence of sex on responses to motivational verbal phrase conditions, change scores between conditions (Δ values) were calculated for each participant, and differences between male and female participants were assessed using the non-parametric Mann–Whitney U test. In addition, Friedman tests were conducted separately within each sex group to examine condition effects; these analyses were exploratory in nature and are presented as supportive findings. All statistical analyses were performed using IBM SPSS Statistics software (version 27.0; IBM Corp., Armonk, NY, United States), and the level of statistical significance was set at *p* < 0.05.

Sample size was determined using an *a priori* power analysis conducted with G*Power software (version 3.1.9.3; Düsseldorf, Germany) (Faul et al., 2007). Power analysis was planned based on a three-condition repeated-measures design in accordance with the experimental protocol. Assuming a medium to large effect size (Cohen’s *f* = 0.38), an alpha level of 0.05, and three measurement conditions, the minimum required sample size was calculated as 30 participants. To compensate for potential participant dropout and data loss, the sample size was increased, and the study was completed with a total of 40 participants, yielding a statistical power of 1 − β = 0.90 (90%). Although non-parametric tests (Friedman and Wilcoxon tests) were used in the final analyses due to incomplete fulfillment of normality assumptions and the ordinal nature of the Borg scale, the *a priori* power analysis was conducted to determine the effect required by the experimental design. Considering the more conservative nature of non-parametric tests, the final sample size was deemed sufficient to meet the objectives of the study. In addition, a previous study employing a similar experimental design and examining the effects of motivational verbal phrases on performance with 28 participants demonstrated that this sample size was adequate to detect acute effects (Van Hooren et al., 2024).

## Results

### Participant characteristics

**Figure 3:**
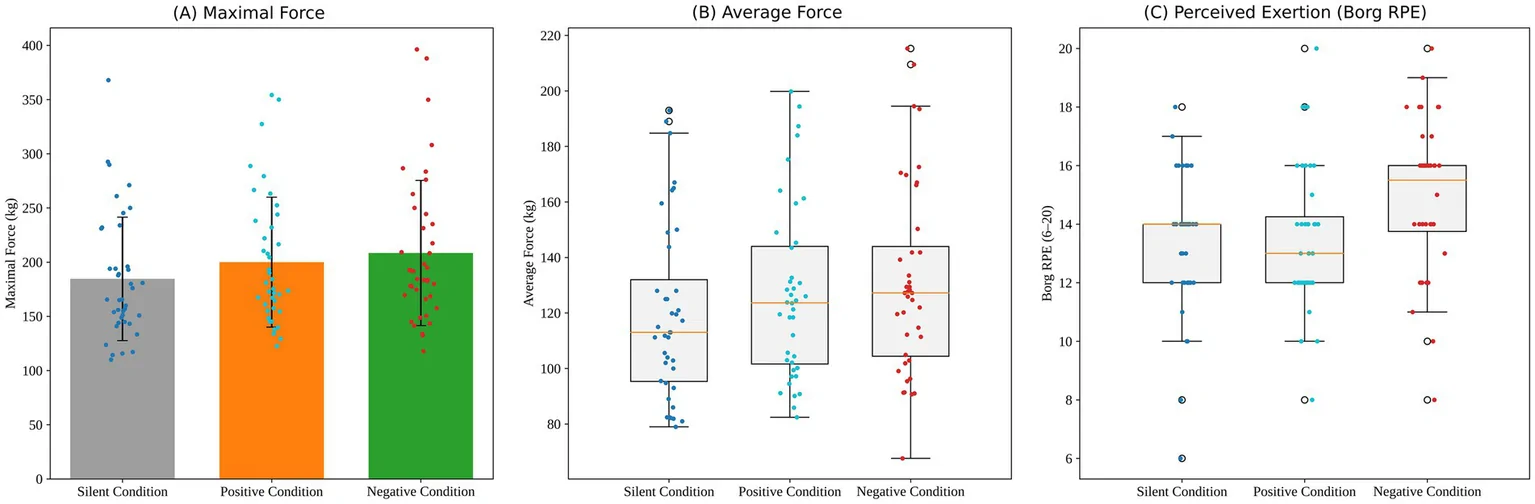
Effects of motivational verbal phrase conditions on isoinertial force production and perceived exertion. **(A)** Maximal force, **(B)** average force, and **(C)** perceived exertion (Borg RPE) across silent, positive, and negative motivational verbal phrase conditions. Bars represent mean ± SD, overlaid points indicate individual participant values. Statistical significance refers to Holm–Bonferroni adjusted pairwise comparisons following Friedman tests *p < 0.05.

A total of 40 sports science students (20 males and 20 females) participated in the study. Male participants had a mean age of 21.50 ± 0.61 years, mean height of 176.25 ± 6.76 cm, mean body weight of 78.55 ± 13.38 kg, and mean BMI of 25.19 ± 3.12 kg/m^2^. Female participants had a mean age of 21.45 ± 1.61 years, mean height of 165.15 ± 4.83 cm, mean body weight of 65.05 ± 15.18 kg, and mean BMI of 23.80 ± 5.16 kg/m^2^. Minimum, maximum, and mean values, along with sex specific descriptive statistics, are presented in Table 2 and Figure 3.

**Table 2:** Descriptive statistics of participants’ demographic characteristics (mean ± SD).

Variable	Age	Height	Weight	BMI
Male	21.50 ± 0.61	176.25 ± 6.76	78.55 ± 13.38	25.19 ± 3.12
Female	21.45 ± 1.61	165.15 ± 4.83	65.05 ± 15.18	23.80 ± 5.16

Supplementary Table S1 presents the results of the Friedman test and *post hoc* Wilcoxon signed-rank tests (Holm-corrected) conducted separately for female and male participants across motivational verbal phrase conditions. For each outcome variable, test statistics, adjusted *p*-values, and corresponding effect size estimates (Kendall’s W) are reported (Tables 3–7).

**Table 3:** Descriptive statistics of outcome variables (mean ± SD).

Variable	Silent	Positive	Negative
Average force (kg)	119.6 ± 31.8	126.8 ± 31.4	131.2 ± 34.7
Maximal force (kg)	184.6 ± 57.0	200.0 ± 59.9	208.4 ± 67.0
Borg scale	13.3 ± 2.4	13.5 ± 2.4	14.8 ± 2.6

**Table 4:** Normality distribution (Shapiro–Wilk Test Results).

Variable	Condition	W	*p*-value	Normal? (*p* > 0.05)
Average force	Silent	0.916	0.006	No
Average force	Positive	0.927	0.013	No
Average force	Negative	0.942	0.039	No
Maximal force	Silent	0.902	0.002	No
Maximal force	Positive	0.898	0.002	No
Maximal force	Negative	0.882	<0.001	No
Borg scale	Silent	0.927	0.012	No
Borg scale	Positive	0.930	0.017	No
Borg scale	Negative	0.961	0.184	Yes

Considering normality and level of measurement, a non-parametric approach (Friedman + Wilcoxon) was used in the main analyses.

**Table 5:** Friedman test results.

Outcome	χ^2^(df = 2)	*p*-value	Kendall’s W
Average force (kg)	31.48	<0.001	0.394
Maximal force (kg)	35.45	<0.001	0.443
Borg scale	10.54	0.005	0.132

The condition effect was significant for both average and maximal force outcomes [Average force: χ^2^(2) = 31.48, *p* < 0.001, *W* = 0.394; Maximal force: χ^2^(2) = 35.45, *p* < 0.001, *W* = 0.443].

**Table 6:** Pairwise comparisons between motivational verbal phrase conditions.

Comparison	*Z*	p (uncorrected)	p (Holm-adjusted)
Average force (kg)			
Silent vs. positive	−3.35	0.00080	0.00080
Silent vs. negative	−4.15	0.000032	0.000096
Positive vs. negative	−3.80	0.000115	0.000231
Maximal force (kg)			
Silent vs. positive	−3.98	0.000067	0.000134
Silent vs. negative	−4.60	0.0000017	0.0000051
Positive vs. negative	−2.85	0.00239	0.00239
Borg scale			
Silent vs. positive	−0.53	0.594	0.594
Silent vs. negative	−3.02	0.00259	0.00778
Positive vs. negative	−2.77	0.00562	0.01125

Pairwise comparisons were performed using the Wilcoxon signed-rank test. Holm–Bonferroni correction was applied to control for multiple comparisons. Statistical significance was set at *p* < 0.05.

**Table 7:** Gender comparison of change scores (Mann–Whitney U).

Outcome	Δ	Male median	Female median	*p*-value
Average force	Δ Positive	7.30	8.60	1.000
Average force	Δ Negative	13.35	9.55	0.218
Maximal force	Δ Positive	18.30	10.00	0.262
Maximal force	Δ Negative	20.95	17.50	0.534
Borg	Δ Positive	0.00	0.00	0.815
Borg	Δ Negative	2.00	2.00	0.612

Mann–Whitney U tests revealed no statistically significant differences between male and female participants in condition-related change scores (Δ values).

## Discussion

The purpose of this study was to compare the effects of positive and negative motivational verbal phrases applied during short term maximal isoinertial squat exercise on force production, average force output across the test duration, and perceived exercise difficulty.

The main findings of the study were as follows: (i) both motivational verbal phrase conditions significantly increased maximal and average force output compared with the silent control condition; (ii) force output was higher under the negative motivational verbal phrase condition than under the positive condition; and (iii) perceived exercise difficulty was significantly greater under the negative motivational verbal phrase condition. These findings partially support the study hypotheses and indicate that not only the presence of motivational verbal phrases, but also their content and emotional valence, play a determining role in performance outcomes.

The effects of motivational verbal phrases on force production have predominantly been examined using isometric and isokinetic tasks. Previous studies have demonstrated that motivational verbal phrases can enhance maximal force, torque, and electromyographic activation (Amagliani et al., 2010; Belkhiria et al., 2018; Campenella et al., 2000). In particular, Belkhiria et al. (2018) reported that motivational verbal phrases applied during a handgrip task increased both force output and muscle activation. Similarly, studies employing isokinetic protocols have shown that motivational verbal phrases lead to significant changes in performance outcomes (Rendos et al., 2019). In the present study, the use of an isoinertial squat protocol extends this body of literature by incorporating a dynamic, functional, and bidirectional loading model, demonstrating that the effects of motivational verbal phrases are not limited to static or machine-based tasks. The present findings are consistent with those of Halperin et al. (2020), who reported greater force production under negative verbal feedback compared with both positive and no feedback conditions during repeated maximal isometric contractions. Although the exercise modalities differed, the direction of the performance response was remarkably similar: in both studies, negatively valenced verbal stimuli elicited the greatest force output. However, whereas Halperin et al. examined repeated isometric elbow flexor contractions and electromyographic activity, the present study extends these findings to a continuous dynamic isoinertial squat involving concentric–eccentric actions. Furthermore, the higher RPE observed under the negative condition in the present study suggests that the additional force output elicited by negatively valenced verbal stimuli may be accompanied by a greater perceptual cost.

Importantly, the influence of motivational verbal phrases on performance outcomes has also been observed beyond strength-based tasks. Recently, Yavuz et al. (2025) reported that, in respiratory function tests conducted in athletes and sedentary individuals, verbal motivation provided during the test compared with motivation delivered prior to testing significantly increased performance indicators such as forced vital capacity (FVC) and peak expiratory flow (PEF). These findings underscore that, in measurement protocols requiring maximal effort, motivational verbal phrases should not be regarded merely as a supportive element, but rather as a critical methodological factor capable of directly influencing measurement outcomes. The elevated force output in response to negative verbal phrases may be attributed to threat induced activation of the sympathy adrenal system, resulting in heightened catecholamine release (e.g., adrenaline and noradrenaline) (Meeusen and De Meirleir, 1995), which improves neuromuscular recruitment and contractile properties (Enoka and Duchateau, 2008; Zatsiorsky and Kraemer, 2006). This “fight-or-flight” response, regulated by the amygdala and the hypothalamic–pituitary–adrenal (HPA) axis (Ulrich-Lai and Herman, 2009), can significantly enhance motor unit recruitment and rate coding. This interpretation aligns with prior studies on stress induced performance enhancement (Dhabhar, 2018; Starcke and Brand, 2012) and indicates that negatively valenced stimuli may function as an acute physiological stressor rather than merely a psychological demotivator (Halperin et al., 2020; Hidalgo et al., 2020).

Previous studies have reported that motivational verbal phrases do not always result in an absolute increase in strength, particularly during tasks associated with substantial fatigue (Lee et al., 2021). However, such verbal stimuli may contribute to the preservation of central motor drive. In this context, the higher average and maximal force values observed under the negative motivational verbal phrase condition in the present study suggest that verbal stimuli can modulate performance through alterations in central nervous system activation, arousal level, attention focus, and threat perception, potentially through heightened threat perception and avoidance related motivational mechanisms. These motivational and perceptual factors represent potential threats to internal validity that are frequently overlooked but should be carefully controlled in exercise science research (Halperin et al., 2015). Consistent with this interpretation, previous research has demonstrated that the source and perceived nature of motivational verbal phrases during functional and high intensity tasks can substantially influence performance outcomes and psychophysiological responses (Romdhani et al., 2024a; Romdhani et al., 2024b). In short duration maximal tasks, negative motivational verbal phrases may elicit heightened neuromotor arousal through threat perception and avoidance related motivation, thereby enhancing force output.

Another notable finding of the present study was the significant increase in perceived exercise difficulty (Borg RPE) under the negative motivational verbal phrase condition. This result aligns with previous studies emphasizing the tradeoff between performance enhancement and perceived effort (Pacholek and Zemková, 2022; Sahli et al., 2024). Research conducted in sports science populations has similarly shown that motivational verbal phrases influence not only performance outcomes but also perceived difficulty and broader psychophysiological responses (Romdhani et al., 2024a; Romdhani et al., 2024b). Accordingly, although negative motivational verbal phrases enhanced force production, they simultaneously increased the subjective perception of effort, suggesting that performance benefits should be interpreted alongside their associated perceptual cost.

The psychophysiological and emotional effects of motivational verbal phrases have been extensively investigated in high intensity tasks such as sprinting, change of direction activities, and small sided games. These studies consistently report that motivational verbal phrases can influence not only performance metrics but also emotional state and perceived exertion (Sahli et al., 2024; Sahli et al., 2020; Sahli et al., 2022). The findings of the present study extend this evidence to isoinertial force tasks, suggesting that the performance enhancing effects of motivational verbal phrases may be independent of specific sport disciplines.

Moreover, the influence of motivational verbal phrases on performance appears not to be restricted to strength-based tasks. Research in other disciplines, including swimming, has demonstrated significant effects of motivational verbal phrases on performance outcomes and muscle fatigue (Puce et al., 2022). Collectively, these findings support the notion that motivational verbal phrases exert multifaceted effects mediated through central and perceptual mechanisms.

With respect to sex-based analyses, no significant differences were observed between male and female participants in their responses to motivational verbal phrase conditions. This finding is consistent with previous studies reporting that the performance effects of motivational verbal phrases are largely independent of sex (Engel et al., 2019; Van Hooren et al., 2024). Consequently, task characteristics, load duration, and the content of motivational verbal phrases appear to be more influential determinants of performance modulation than sex related factors.

## Conclusion

This study is among the limited number of investigations examining the effects of motivational verbal phrases applied during maximal isoinertial squat exercise on force production, sustained force output across the test duration, and perceived exercise difficulty. The findings indicate that both positive and negative motivational verbal phrases increased force production compared with the silent control condition; however, negative motivational verbal phrases elicited a more pronounced enhancement in force output. In contrast, perceived exercise difficulty was also significantly higher under negative motivational verbal phrase conditions. These results demonstrate that not only the presence but also the content and emotional tone of motivational verbal phrases are critical determinants of both performance and perceptual responses. The deliberate and goal-oriented use of motivational verbal phrases within training and performance contexts may therefore represent an effective strategy for enhancing short term maximal performance.

## Limitations and future directions

Several limitations of the present study should be acknowledged. First, the standardized motivational verbal phrase protocol applied under laboratory conditions may not fully capture the complexity of verbal stimuli encountered in real sporting environments, where athletes may simultaneously receive verbal input from coaches, teammates, opponents, and spectators.

Second, the study employed an acute experimental design, focusing exclusively on the short-term effects of motivational verbal phrases. Consequently, the long-term effects of negative motivational verbal phrases on training adaptations, motivation, and psychological well-being could not be examined and warrant further investigation.

Third, perceived exercise difficulty was assessed using the Borg RPE scale; however, affective responses, physiological stress markers, and neurophysiological indices (e.g., electromyography or measures of central motor activation) were not directly evaluated. The inclusion of such variables in future studies may provide a more comprehensive understanding of the relationship between performance enhancement and perceptual responses.

Moreover, although participants were instructed to exert the greatest possible effort throughout the 30-s test, the mean RPE values of approximately 14.5–14.8 corresponded to a hard, but not maximal, level of perceived exertion. Therefore, these RPE values should not be interpreted as evidence that a physiologically maximal response was attained. Rather, they represent participants’ overall perception of effort under each verbal phrase condition. The higher RPE observed in the discouraging condition indicates greater perceived task demand relative to the other conditions, but does not independently verify maximal performance.

Fourth, while negative verbal phrases enhanced acute force output, this study did not examine the long-term effects of repeated exposure to aversive verbal stimuli. Prolonged or frequent use of negative verbal encouragement in training contexts may lead to increased burnout, reduced intrinsic motivation, and impaired psychological well-being, consistent with Self-Determination Theory’s emphasis on autonomy and competence support. Future longitudinal studies should investigate the tradeoff between acute performance enhancement and chronic motivational costs.

The absence of validated psychological assessments represents an additional limitation of this study. Because questionnaires assessing characteristics such as motivation, self-efficacy, competitiveness, perceived threat, and personality were not administered, participants could not be classified according to psychological profiles that may have influenced their responses to encouraging and discouraging verbal phrases. Future studies should incorporate validated psychological measures to examine whether these characteristics moderate the effects of verbal phrases on exercise performance and perceived exertion.

Finally, the sample consisted of healthy young adults, which may limit the generalizability of the findings to elite athletes, younger populations, or individuals with different performance levels. Future research should examine the effects of motivational verbal phrases across diverse populations and sporting disciplines.

## Practical implications

Based on the findings of the present study, several practical implications can be proposed. Coaches and practitioners should recognize that motivational verbal phrases represent a powerful tool for modulating performance, with the content of these phrases playing a critical role in determining outcomes. Negative motivational verbal phrases may be used in a limited and controlled manner to enhance performance during tasks requiring short term maximal force output.

Conversely, the prolonged or repeated use of negative motivational verbal phrases during routine training sessions or high-volume workloads may increase perceived exertion and potentially lead to unfavorable psychological and physiological consequences. In such contexts, the use of positive or neutral motivational verbal phrases may be more appropriate.

Additionally, the standardization of motivational verbal phrases in both training and testing environments may improve the reliability of performance assessments. Finally, the development of individualized verbal phrase strategies tailored to athletes’ personal characteristics, motivational orientations, and responsiveness to verbal feedback is recommended to optimize performance outcomes.

## Data Availability

The raw data supporting the conclusions of this article will be made available by the authors, without undue reservation.

## Footnotes

1
www.randomizer.org
