# Supplementary material for: The effects of motivational verbal phrases on isoinertial force production and sustained force output: a comparison of positive and negative conditions
Source: Front Psychol. 2026 Sep 10;17:1852572. doi: 10.3389/fpsyg.2026.1852572 (PMC13600893; doi:10.3389/fpsyg.2026.1852572)
Supplement: Supplementary file 1 [file Data_Sheet_1.docx]

**Supplementary Material S1.**

**Randomization**

A randomized, counterbalanced crossover design was employed. Each participant completed three experimental sessions, during which all three verbal conditions (silent control, positive verbal encouragement, and negative verbal encouragement) were administered once.

Participants were not informed about the total number of sessions or the type of verbal condition to be applied in each session, ensuring a single-blind design.

For the first session, both the order of participant testing and the assigned verbal condition were determined randomly. In the second session, participants were again tested in a newly randomized order and assigned randomly to one of the two remaining conditions not yet completed. In the third session, participants completed the final remaining condition, with the testing order randomized once more.

To minimize potential carry-over effects, sessions were separated by a minimum of 48 hours.

Randomization sequences were generated individually for each participant using a computer-based randomization tool ([www.randomizer.org](https://www.randomizer.org)). All participants were exposed to each condition; however, the order of condition exposure was counterbalanced across participants.

Detailed randomization sequences

| **1. Session Ordering and Conditions** | | | **2. Session Ordering and Conditions** | | | **3. Session Ordering and Conditions** | | |
| --- | --- | --- | --- | --- | --- | --- | --- | --- |
| (5, 28, 36, 2, 11, 18, 37, 31, 25, 9, 8, 7, 12, 39, 38, 15, 33, 20, 16, 29, 3, 6, 34, 14, 24, 17, 35, 27, 10, 26, 13, 4, 23, 19, 30, 21, 40, 32, 22, 1) | | | (33, 11, 6, 7, 2, 27, 22, 19, 36, 4, 12, 38, 37, 25, 21, 35, 10, 17, 29, 16, 13, 3, 9, 8, 14, 26, 24, 20, 40, 1, 31, 5, 15, 32, 30, 18, 34, 39, 23, 28) | | | (6, 32, 14, 21, 9, 40, 20, 36, 7, 19, 22, 30, 31, 27, 25, 15, 4, 23, 26, 3, 2, 13, 24, 8, 1, 34, 10, 29, 12, 5, 11, 38, 39, 18, 16, 17, 37, 33, 28, 35) | | |
| **Participant** | **Rank** | **Condition** | **Participant** | **Rank** | **Condition** | **Participant** | **Rank** | **Condition** |
| 1 | 5 | Neutral | 1 | 33 | Negative | 1 | 6 | Positive |
| 2 | 28 | Positive | 2 | 11 | Positive | 2 | 32 | Neutral |
| 3 | 36 | Negative | 3 | 6 | Neutral | 3 | 14 | Neutral |
| 4 | 2 | Negative | 4 | 7 | Neutral | 4 | 21 | Positive |
| 5 | 11 | Neutral | 5 | 2 | Positive | 5 | 9 | Positive |
| 6 | 18 | Negative | 6 | 27 | Negative | 6 | 40 | Neutral |
| 7 | 37 | Positive | 7 | 22 | Negative | 7 | 20 | Neutral |
| 8 | 31 | Positive | 8 | 19 | Neutral | 8 | 36 | Positive |
| 9 | 25 | Positive | 9 | 36 | Neutral | 9 | 7 | Negative |
| 10 | 9 | Neutral | 10 | 4 | Negative | 10 | 19 | Negative |
| 11 | 8 | Negative | 11 | 12 | Neutral | 11 | 22 | Neutral |
| 12 | 7 | Positive | 12 | 38 | Positive | 12 | 30 | Positive |
| 13 | 12 | Negative | 13 | 37 | Neutral | 13 | 31 | Negative |
| 14 | 39 | Neutral | 14 | 25 | Neutral | 14 | 27 | Positive |
| 15 | 38 | Negative | 15 | 21 | Negative | 15 | 25 | Negative |
| 16 | 15 | Neutral | 16 | 35 | Positive | 16 | 15 | Positive |
| 17 | 33 | Neutral | 17 | 10 | Negative | 17 | 4 | Neutral |
| 18 | 20 | Negative | 18 | 17 | Positive | 18 | 23 | Negative |
| 19 | 16 | Positive | 19 | 29 | Positive | 19 | 26 | Neutral |
| 20 | 29 | Neutral | 20 | 16 | Negative | 20 | 3 | Positive |
| 21 | 3 | Neutral | 21 | 13 | Neutral | 21 | 2 | Neutral |
| 22 | 6 | Negative | 22 | 3 | Negative | 22 | 13 | Negative |
| 23 | 34 | Positive | 23 | 9 | Negative | 23 | 24 | Positive |
| 24 | 14 | Negative | 24 | 8 | Positive | 24 | 8 | Neutral |
| 25 | 24 | Negative | 25 | 14 | Positive | 25 | 1 | Negative |
| 26 | 17 | Neutral | 26 | 26 | Positive | 26 | 34 | Neutral |
| 27 | 35 | Neutral | 27 | 24 | Neutral | 27 | 10 | Neutral |
| 28 | 27 | Neutral | 28 | 20 | Positive | 28 | 29 | Negative |
| 29 | 10 | Positive | 29 | 40 | Negative | 29 | 12 | Positive |
| 30 | 26 | Negative | 30 | 1 | Neutral | 30 | 5 | Negative |
| 31 | 13 | Positive | 31 | 31 | Neutral | 31 | 11 | Negative |
| 32 | 4 | Positive | 32 | 5 | Positive | 32 | 38 | Neutral |
| 33 | 23 | Neutral | 33 | 15 | Negative | 33 | 39 | Positive |
| 34 | 19 | Positive | 34 | 32 | Positive | 34 | 18 | Positive |
| 35 | 30 | Negative | 35 | 30 | Neutral | 35 | 16 | Neutral |
| 36 | 21 | Neutral | 36 | 18 | Neutral | 36 | 17 | Neutral |
| 37 | 40 | Positive | 37 | 34 | Negative | 37 | 37 | Negative |
| 38 | 32 | Negative | 38 | 39 | Negative | 38 | 33 | Positive |
| 39 | 22 | Positive | 39 | 23 | Positive | 39 | 28 | Neutral |
| 40 | 1 | Positive | 40 | 28 | Negative | 40 | 35 | Negative |
